# Nutraceutical Profiles of Two Hydroponically Grown Sweet Basil Cultivars as Affected by the Composition of the Nutrient Solution and the Inoculation With *Azospirillum brasilense*

**DOI:** 10.3389/fpls.2020.596000

**Published:** 2020-11-05

**Authors:** Simun Kolega, Begona Miras-Moreno, Valentina Buffagni, Luigi Lucini, Fabio Valentinuzzi, Mauro Maver, Tanja Mimmo, Marco Trevisan, Youry Pii, Stefano Cesco

**Affiliations:** ^1^Faculty of Science and Technology, Free University of Bozen-Bolzano, Bolzano, Italy; ^2^Department of Ecology, Agronomy and Aquaculture, University of Zadar, Zadar, Croatia; ^3^Department for Sustainable Food Process, Università Cattolica del Sacro Cuore, Piacenza, Italy; ^4^Competence Centre for Plant Health, Free University of Bozen/Bolzano, Bolzano, Italy

**Keywords:** basil (*Ocimum basilicum* L.), metabolomics, ionomics, *Azospirillum brasilense* Cd, fertilization strategies, hydroponics and soilless culture, nutraceuticals

## Abstract

Sweet basil (*Ocimum basilicum* L.) is one of the most produced aromatic herbs in the world, exploiting hydroponic systems. It has been widely assessed that macronutrients, like nitrogen (N) and sulfur (S), can strongly affect the organoleptic qualities of agricultural products, thus influencing their nutraceutical value. In addition, plant-growth-promoting rhizobacteria (PGPR) have been shown to affect plant growth and quality. *Azospirillum brasilense* is a PGPR able to colonize the root system of different crops, promoting their growth and development and influencing the acquisition of mineral nutrients. On the bases of these observations, we aimed at investigating the impact of both mineral nutrients supply and rhizobacteria inoculation on the nutraceutical value on two different sweet basil varieties, i.e., *Genovese* and *Red Rubin*. To these objectives, basil plants have been grown in hydroponics, with nutrient solutions fortified for the concentration of either S or N, supplied as SO_4_^2–^ or NO_3_^–^, respectively. In addition, plants were either non-inoculated or inoculated with *A. brasilense*. At harvest, basil plants were assessed for the yield and the nutraceutical properties of the edible parts. The cultivation of basil plants in the fortified nutrient solutions showed a general increasing trend in the accumulation of the fresh biomass, albeit the inoculation with *A. brasilense* did not further promote the growth. The metabolomic analyses disclosed a strong effect of treatments on the differential accumulation of metabolites in basil leaves, producing the modulation of more than 400 compounds belonging to the secondary metabolism, as phenylpropanoids, isoprenoids, alkaloids, several flavonoids, and terpenoids. The primary metabolism that resulted was also influenced by the treatments showing changes in the fatty acid, carbohydrates, and amino acids metabolism. The amino acid analysis revealed that the treatments induced an increase in arginine (Arg) content in the leaves, which has been shown to have beneficial effects on human health. In conclusion, between the two cultivars studied, *Red Rubin* displayed the most positive effect in terms of nutritional value, which was further enhanced following *A. brasilense* inoculation.

## Introduction

Basil (*Ocimum basilicum* L.) is an annual plant belonging to the *Lamiaceae* family growing in the tropical and subtropical regions of America, Africa, Asia, and the southern areas of Europe (Kwee and Niemeyer, 2011). Basil has considerable commercial importance not only as a fresh-market herb for culinary and ornamental purposes but also for the production of phytochemicals used for medicinal scopes (Singletary, 2018). The medicinal properties of basil can be mainly ascribed to the presence of a plethora of biologically active compounds in its leaves, characterized by different chemical structure, encompassing for instance phenolic acids (i.e., rosmarinic and caffeic acids), flavonol glycosides (quercetin and kaempferol), and anthocyanins (Flanigan and Niemeyer, 2014; Ghasemzadeh et al., 2016; Złotek et al., 2016; Singletary, 2018). Other important components contained in both basil leaves and flowers are essential oils, which play a pivotal role in the medicinal and food application of this plant (Avetisyan et al., 2017; Burducea et al., 2018).

The qualitative and quantitative composition of phytochemicals featured by basil leaves can primarily depend on plant’s genetic traits (Skrypnik et al., 2019). Indeed, several authors have shown that distinct basil cultivars have the genetic potential of generating and storing different sets of bioactive molecules, thus leading to a wide range of possible chemotypes within the same basil variety/species (Avetisyan et al., 2017). Besides genetics, another critical factor determining the set of phytochemicals produced by basil, and by plants in general, is the cultivation condition (Scagel and Lee, 2012). The cultivation of basil plants is normally carried out in both natural (e.g., open field) and controlled conditions (e.g., greenhouses); however, to increase the yield in terms of biomass as well as to prolong the production period over the year, the greenhouse cultivation methods represent the most suitable solution (Sgherri et al., 2010). Concerning the growth in controlled conditions, at present, the hydroponic cultivation of basil is the preferred solution as compared to the traditional soil-based cultivation methods (Kiferle et al., 2011). Indeed, the soilless cultivation approach represents a good opportunity for agriculture, especially for those areas that are characterized by scarce water availability and severe soil degradation, allowing the implementation of environment-friendly agricultural practices in a general context of safe food production (Sambo et al., 2019). The exploitation of controlled conditions allowed by the hydroponic cultivation methods consents, on the one hand, the reduction in soil disinfection and application of agrochemicals with defense purposes and, on the other hand, the fine tuning of the nutrient solutions compositions to match plants nutritional requirements to maximize both the yield and the quality of the agricultural products (Sambo et al., 2019). In this sense, hydroponic cultivation systems also allow a better reproducibility in plant growth and yield as well as in the quality of the agricultural products in term of nutraceuticals content (Kiferle et al., 2011; Sambo et al., 2019; Skrypnik et al., 2019).

The content of bioactive compounds in basil leaves can be dependent on both the growth substrate and the fertilizer (e.g., chemical vs. organic) applied (Matłok et al., 2019). In particular, mineral nutrition has been addressed as one of the principal features influencing plant metabolism. Several authors, for instance, have highlighted that the fertilization with the essential macronutrients potassium (K^+^) and ammonium (NH_4_^+^) can lead to an increased content of nutraceutical compounds, like sugars, phenolic acids, flavonoids, anthocyanins, carotenoids, lycopene, and vitamins, in both medicinal herbs like basil and fruits (Lester et al., 2010; Ibrahim et al., 2012; Salas-Pérez et al., 2018; Valentinuzzi et al., 2018b,c). Few pieces of research also indicate that the overfertilization with sulfur (S) can induce a higher yield in several plants, as for instance *Medicago sativa*, wheat, canola, oil seed rape, corn, and potato (Stewart and Porter, 1969; Mullins and Mitchell, 1989; Weil and Mughogho, 2000; Pavlista, 2005; Rehm, 2005); in the specific case of basil plants, sulfate (SO_4_^2–^) increased fertilization rate and resulted in a higher accumulation of biomass and a higher concentration of eucalyptol in the leaves (Zheljazkov et al., 2008). Interestingly, several experimental experiences also showed that the biofortification practices with non-essential microelements [e.g., boron (B), silicon (Si), and selenium (Se)] can produce an alteration in the metabolomic profiles of agricultural products, inducing the accumulation of secondary metabolites, which might have beneficial effects on human health (Gottardi et al., 2012; Schiavon et al., 2013; Nancy and Arulselvi, 2014; Tomasi et al., 2015; Mimmo et al., 2017; Valentinuzzi et al., 2018a,c; Skrypnik et al., 2019). In fact, the preventive effects arising from the consumption of fruit, vegetables, and herbs toward chronic diseases are mostly due to secondary metabolites such as vitamins (including carotenoids and tocopherols) and antioxidant compounds such as glucosinolates and phenolic compounds (Schreiner et al., 2012). While primary metabolites are ubiquitous in plant species, the products of the secondary metabolism, which include the majority of the industrially applicable compounds, represent a complex adaptation mechanism that plants adopt to face adverse environmental conditions (Isah, 2019). In this vision, the application of abiotic stressors (e.g., mild nutrient deficiencies, UV light) has been proposed as an alternative strategy to enhance the content of nutraceuticals compounds in agricultural products for human consumption (Kiferle et al., 2011; Valentinuzzi et al., 2015; Burducea et al., 2018), albeit this method might have a negative impact on the cultivation yield. Beside the modulation of the growing media composition, additional strategies, including high-yielding cell line screening, elicitation, precursor feeding, large-scale cultivation systems, plant cell immobilization, hairy root culture, and biotransformation (Ramachandra Rao and Ravishankar, 2002; Cardoso et al., 2019), aimed at increasing the content of technologically relevant secondary metabolites in medicinal and edible plants, have been explored.

In this sense, it is widely known that a group of bacteria, generally known as plant growth-promoting rhizobacteria (PGPR), can positively affect the growth and the fitness of plants as well as crop yield and quality (Pii et al., 2015b). The positive effects exerted by PGPR on plants can be brought about through both direct (e.g., inducing the growth of root system, assimilating atmospheric inorganic nitrogen, increasing the bioavailability of mineral nutrients, and enhancing plants ability to take up mineral nutrients) and indirect (e.g., activating plants induced systemic resistance, producing antimicrobial compounds, and outcompeting pathogens for essential nutrients) mechanisms (Pii et al., 2015b; Crecchio et al., 2018). Nonetheless, the interaction between PGPR and plant roots has also been shown to induce modulation in the molecular and biochemical mechanisms related to different aspects of plant physiology, thus having an impact on the production and content of secondary metabolites. Several pieces of evidence have already addressed the positive role produced by PGPR inoculation on the quality of fruits like citrus, mulberry, apricot, sweet cherry, raspberry, and strawberry (Esitken et al., 2002, 2003, 2005, 2006; Orhan et al., 2006; Pii et al., 2018). More recently, PGPR as well as symbiotic arbuscular mycorrhizal fungi have been shown to have a promoting effect on the accumulation of secondary metabolites, like antioxidant molecules and essential oils, in aromatic herbs (Banchio et al., 2009; Erika et al., 2010; Santoro et al., 2011; Heidari and Golpayegani, 2012; Cappellari et al., 2013, 2019a,b). Nonetheless, the effect produced by the rhizobacteria on the qualitative and quantitative profile of secondary metabolites has been observed to be strongly dependent both on the plant species/cultivar and on the microbial strain. This suggests the existence of specific microbe/host recognition and interaction mechanisms, thus inducing diverse effects at plant level (Cappellari et al., 2019a).

On the bases of these premises, the aim of this work was to assess the influence of the mineral nutrients supply, as well as the inoculation of the PGPR *Azospirillum brasilense*, on the nutraceutical compounds production in two different sweet basil cultivars, namely, *Genovese* and *Red Rubin*. For the purposes of this study, the macronutrients nitrogen (N) and sulfur (S) were selected for the fortification of the nutrient solutions, mainly for the presence of these elements in the chemical structure of secondary metabolites with nutraceutical value. To this aim, basil plants were hydroponically grown with two nutrient solutions, fortified for the concentration of either S, supplied as SO_4_^2–^, or N, supplied as NO_3_^–^. In addition, plants were inoculated with *A. brasilense*, which is already known (i) to influence the molecular and biochemical mechanisms underlying the nutrients acquisition in model plants (Pii et al., 2015c, 2016, 2019; Marastoni et al., 2019) and (ii) determine alteration in both quantitative and qualitative traits of strawberry fruits (Pii et al., 2018). The effects of cultivation practices (i.e., nutrient solution fortification and inoculation with beneficial microbes) on the nutraceutical composition in basil leaves have been assessed by the application of a holistic approach, combining metabolomics and ionomic analyses, together with traditional analytical methods, based on both spectrophotometric and high-performance liquid chromatography (HPLC) analyses.

## Materials and Methods

### Plant Material and Growing Conditions

The seeds of two different sweet basil (*Ocimum basilicum*) cultivars (i.e., cv. *Genovese* and cv. *Red Rubin*) were obtained from a local nursery and were germinated adopting a modified version of the RHIZOtest system (Bravin et al., 2010). Seeds were sown in small pots and placed in 6-L plastic tanks. For the germination stage, tanks were filled with 6 L of germination solution (CaCl_2_, 88.24 g L^–1^; H_3_BO_3_, 0.1236 g L^–1^) and covered with both plastic and aluminum foils (Bravin et al., 2010). Air pumps were used to maintain the constant aeration of the nutrient solution. Plants have been grown in controlled conditions in a climatic chamber with a day/night cycle of 14/10 h and 24/19°C. The relative humidity was about 70%, and the light intensity was 250 mmol m^–2^ s^–1^. One week after sowing, germination solution was replaced with nutrient solution (NS), composed of the following: 14.58 mM NO_3_^–^, 3 mM NH_4_^+^, 3.5 mM PO_4_^3–^, 10.5 mM K^+^, 3.5 mM Mg^2+^, 4 mM Ca^2+^, 3.63 mM SO_4_^2–^, 0.95 mM Cl^–^, 40 μM FeEDTA, 10 μM MnSO_4_, 5 μM ZnSO_4_, 1 μM CuSO_4_, 40 μM H_3_BO_3_, and 0.5 μM Na_2_MoO_4_ (pH 6). The control solution has been renewed two times per week for 3 weeks.

After 21 days of growing, samples were split in three sets: one set was used as control and was grown in control NS, mentioned above. The other two sets of samples were grown in modified NS (Supplementary Table 1), containing either 20 mM NO_3_^–^ or 8 mM SO_4_^2–^, while micronutrient concentration was maintained as described above. In addition, half of all the samples were inoculated with the PGPR *A. brasilense*. Six independent biological replicates for each treatment were set up. Basil plants were grown in modified nutrient solutions for a further 3 weeks. At harvest, the soil–plant analysis development (SPAD) measures were recorded, roots and shoots were separated, and fresh weight were recorded. The remaining tissues were frozen in liquid nitrogen and stored in aluminum foils at −80°C for further analyses.

### Bacterial Strain and Inoculation

*A. brasilense* Cd (DSM-1843) was grown in solid Luria–Bertani (LB) growth medium (10 g L^–1^ tryptone, 5 g L^–1^ yeast extract, 10 g L^–1^ NaCl, 14 g L^–1^ agar) for 3 days at 28°C. Afterward, the microbial biomass was inoculated in liquid LB medium and grown at 28°C under horizontal shaking until saturation. Bacteria were collected by centrifugation for 15 min at 4,500 rpm and washed once with sterile deionized water, as previously described (Pii et al., 2019). The concentration of bacteria was estimated by a spectrophotometer at 600 nm optical density. The final concentration of 10^6^ cfu ml^–1^ was used for nutrient solution. A second inoculation was carried out 10 days after the first one.

### Extracts Preparation

Basil leaf extracts were prepared as previously described (Valentinuzzi et al., 2015). Briefly, the tissue was freeze dried and finely homogenized by a ball mill (MM400, Retsch, Italy). A specific amount of powder was weighed into 15-ml centrifuge tubes and resuspended with methanol in a 1:10 ratio. The suspension was thoroughly mixed and sonicated in an ultrasonic bath sonicator for 30 min and centrifuged for 30 min at 4,500 rpm. The supernatants were filtered with a 0.45 μm Nylon syringe filter in 5 ml Eppendorf tubes and kept at −80°C until further analyses.

### Phenolic Acids Analysis

The content of caffeic and rosmarinic acid in leaves was determined as previously described (Kiferle et al., 2011). Briefly, the analyses were performed by using a Waters Alliance HPLC system equipped with a LiChrospher RP-18 column 250 mm × 4.6 mm, 5 μm (Phenomenex, United States), and the elution was carried out at a flowrate of 1.0 mL min^–1^ at room temperature. Two mobile phases, A and B, were used: mobile phase A was acetonitrile, while mobile phase B was 0.1% phosphoric acid. The gradient used was 0–4 min, B 95%; 4–5 min, B 95–85%; 5–10 min, B 85–80%; 10–20 min, B 80–60%; 20–21 min, B 60–5%; 21–25 min, B 5%; 25–26 min, B 5–95%; and 26–30 min, B 95%. The volume injected was 20 μl, and the UV absorption was monitored at 325 nm.

### Analysis of Amino Acids

Amino acids (AAs) were separated by HPLC prior precolumn derivatization with a commercial kit (AccQ∙Tag, WAT052880-Waters Corporation, Italy) using a high-efficiency Nova-PakTMC18 silica-based bonded column (4.6 × 250 mm, 4 mm, Waters Corporation, Italy), with a gradient elution with a flowrate of 1.0 ml/min: A = AccQ∙Tag Eluent (WAT052890), B = 100% acetonitrile (HPLC grade), C = Milli-Q (Waters application note for AA). The derivatized samples were detected using fluorescence detection (λex = 250 nm, λems = 395 nm, Waters 2475, Italy) with the column condition set at 37°C.

### Elements Analyses

Lyophilized and ground basil shoot samples were mineralized with ultrapure 68% HNO_3_ (Carlo Erba) using a single-reaction chamber microwave (SRC, UltraWAVE, Milestone Inc., Shelton, CT, United States). Subsequently, mineralized samples were filtered and elements concentration determined by inductively coupled plasma–optical emission spectrometry (ICP-OES) (Arcos Ametek, Spectro, Germany), using tomato leaves (SRM 1573a) and spinach leaves (SRM 1547) as external certified reference material.

### Total Nitrate Content

Total nitrate content was determined using the methodology described previously (Cataldo et al., 1975). Briefly, 0.1 g of leaves was suspended in 10 ml of distilled water, thoroughly mixed and kept at 45°C for 1 h. Afterward, samples were filtered through Whatman No. 40 filter paper and analyzed immediately. Samples (0.2 ml) were added to 0.8 ml of 5% salicylic acid in concentrated sulfuric acid and incubated for 20 min at room temperature; then, 19 ml of 2 N NaOH was added, and the samples were incubated for further 30 min. The absorbance was read at 410 nm, and the concentration of NO_3_^–^ in the tissue was determined through a suitable calibration curve.

### Untargeted Metabolomics Profiling

Six independent biological replicates from each treatment were analyzed using a ultra-HPLC (UHPLC) system coupled to a hybrid quadrupole-time-of-flight mass spectrometer (UHPLC/QTOF-MS) as previously reported (Rocchetti et al., 2018b). The apparatus comprised a 1290 LC system coupled to a G6550 QTOF detector equipped with an electrospray ionization source (all from Agilent Technologies, Santa Clara, CA, United States). A volume of 4 μl was injected; then, metabolites were separated in reverse phase mode by an Agilent Zorbax Eclipse-plus C18 column (100 mm × 2.1 mm, 1.8 μm), using linear binary gradient elution (6–94% methanol, 33 min run time, flowrate of 200 μl min^–1^). The mass spectrometer operated in SCAN mode (100–1,000 m/z, 0.8 spectra/s) and positive polarity.

Features were extracted and annotated from raw data using Profinder B.07 (Agilent Technologies) following alignment, by merging the monoisotopic accurate mass and the isotopic profile (isotope spacing and ratio). The process was carried out as previously described (Rocchetti et al., 2018a), using the publicly available databases PlantCyc 12.6 (Plant Metabolic Network^1^, downloaded on April 2018) and adopting a mass accuracy tolerance of 5 ppm. On this basis, a putative Level 2 annotation was achieved, as referred by COSMOS Metabolomics Standards^2^. Annotated features were finally filtered by frequency to retain compounds being in at least 75% of replicates within at least one treatment.

### Statistical Analysis

The results are reported as mean ± standard error (SE) of six independent biological replicates. The significance of differences among means was calculated by one-way ANOVA with *post hoc* Tukey honestly significant difference (HSD) with α = 0.05 using R software (version 3.6.0). The following R packages were used for data visualization and statistical analyses: ggplot2 v.3.2.0 (Wickham, 2016), Agricolae v.1.3-1 (de Mendiburu and de Mendiburu, 2019), and ggfortify (Tang et al., 2016).

For the metabolomics analysis, the chemometric interpretations were performed in Mass Profiler Professional B.12.06 as previously reported (Salehi et al., 2018) for log2 transformation of compounds abundance and normalization at the 75th percentile, using the median value as the baseline. Unsupervised hierarchical cluster analysis (HCA), based on fold change (FC) values, was then performed (Euclidean distance matrix, Wards’ linkage) to point out the relatedness of metabolomic signatures across treatments. After that, the supervised orthogonal projections to latent structures discriminant analysis (OPLS-DA) was carried out in SIMCA 13 (Umetrics, Sweden). Outliers were investigated using Hotelling’s T2 (95 and 99% confidence limits for the suspect and strong outliers, respectively). CV-ANOVA (*p* < 0.01) and permutation testing (*N* = 100) were used for model validation and for excluding overfitting, respectively. Goodness-of-fit R2Y and goodness-of-prediction Q2Y were also calculated from the OPLS-DA model. Subsequently, the most discriminant compounds (VIP score > 1.3) were selected by variable importance in projection (VIP) analysis and discriminant compounds identified by Volcano plot analysis, combining fold-change (FC > 1.5) and ANOVA [*p* < 0.05, false discovery rate (FDR) multiple testing correction] to describe the extent and direction of regulation in response to treatments. Finally, the metabolites identified by Volcano analysis along with their FC values were uploaded to the PlantCyc pathway Tools software (Karp et al., 2010) to decipher principal classes of functional compounds modulated by the treatments.

## Results

### Biometric Parameters

**FIGURE 1:**
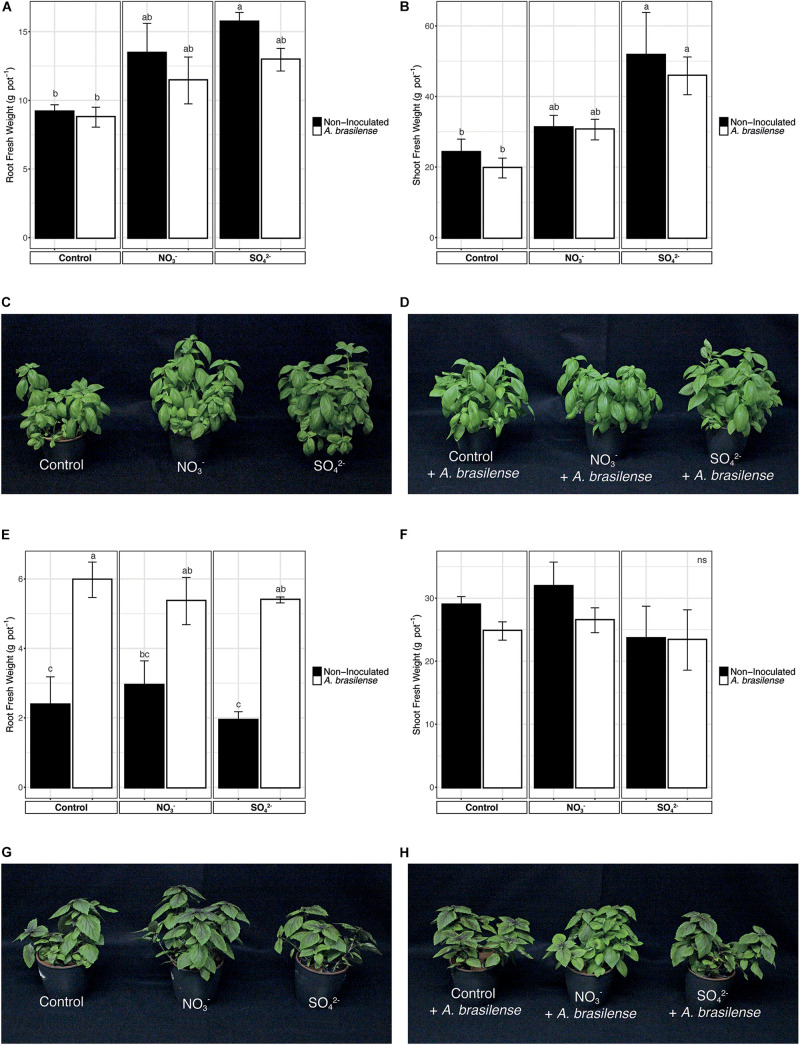
Fresh biomasses of sweet basil plants at the end of the growing period. **(A)** Root biomass of cv. *Genovese* plants grown in control hydroponic solution, in a NO_3_^–^ or in a SO_4_^2–^ overfertilized nutrient solution, either non-inoculated or inoculated with *A. brasilense*. (**B**) Shoot biomass of cv. *Genovese* plants grown in control hydroponic solution, in a NO_3_^–^ or in a SO_4_^2–^ overfertilized nutrient solution, either non-inoculated or inoculated with *A. brasilense*. **(C)** Representative pictures of sweet basil cv. *Genovese* plants grown in control hydroponic solution, in a NO_3_^–^ or in a SO_4_^2–^ overfertilized nutrient solution. **(D)** Representative pictures of sweet basil cv. *Genovese* plants grown in control hydroponic solution, in a NO_3_^–^- or in a SO_4_^2–^-overfertilized nutrient solution and inoculated with *A. brasilense*. **(E)** Root biomass of cv. *Red Rubin* plants grown in control hydroponic solution, in a NO_3_^–^ or in a SO_4_^2–^ overfertilized nutrient solution, either non-inoculated or inoculated with *A. brasilense*. **(F)** Shoot biomass of cv. *Red Rubin* plants grown in control hydroponic solution, in a NO_3_^–^ or in a SO_4_^2–^ over-fertilized nutrient solution, either non-inoculated or inoculated with *A. brasilense*. **(G)** Representative pictures of sweet basil cv. *Red Rubin* plants grown in control hydroponic solution, in a NO_3_^–^ or in a SO_4_^2–^ overfertilized nutrient solution. **(H)** Representative pictures of sweet basil cv. *Red Rubin* plants grown in control hydroponic solution, in a NO_3_^–^ or in a SO_4_^2–^ overfertilized nutrient solution and inoculated with *A. brasilense*. Data are reported as means ± SE, *n* = 6. The statistical significance was tested by means of ANOVA with Tukey posttest. Different letters indicate statistically different values (*p* < 0.05).

At harvest, the fresh weight of the roots and shoot of basil plants, both *Genovese* and *Red Rubin* cultivars, was assessed (Figure 1). In the case of cv. *Genovese*, the root biomass of plants, independently from the nutrient solution (NS) applied, was not affected by the inoculation with *A. brasilense* (Figure 1A). Nonetheless, the increased concentration of S in the NS induced a higher growth of the root biomass in non-inoculated plants as compared to the control ones (Figure 1A). A similar behavior was also observed at shoot level; in fact, the inoculation with the PGPR did not influence the allocation of biomass, whereas the fertilization with SO_4_^2–^ caused a significant increase in the weight of the aerial parts (Figures 1B–D). The cv. *Red Rubin*, though, was differently affected by treatments as compared to cv. *Genovese* (Figure 1). In fact, at the root level, the inoculation with *A. brasilense* caused a strong increase in the biomass (almost doubled) as compared to non-inoculated plants, irrespective of the NS applied (Figure 1E). On the other hand, the overfertilization with either NO_3_^–^ or SO_4_^2–^ did not cause an alteration in the root biomass allocation as compared to control plants (Figure 1E). Yet, the shoot biomass of *O. basilicum* cv. *Red Rubin* was not significantly affected by treatments as compared to control plants (Figures 1F–H).

In addition, the general health status of plants was checked by assessing the total chlorophyll content of leaves that was determined by measuring the SPAD index. The data reported in Supplementary Figure 1 were recorded after 6 weeks of cultivation in hydroponic solution and show that, for both *Genovese* and Red Rubin cultivars, the treatments imposed have no effects on the whole content of chlorophyll in the leaves of sweet basil (Supplementary Figure 1).

## Leaves Quality Assessment

### Untargeted Metabolomics

**FIGURE 2:**
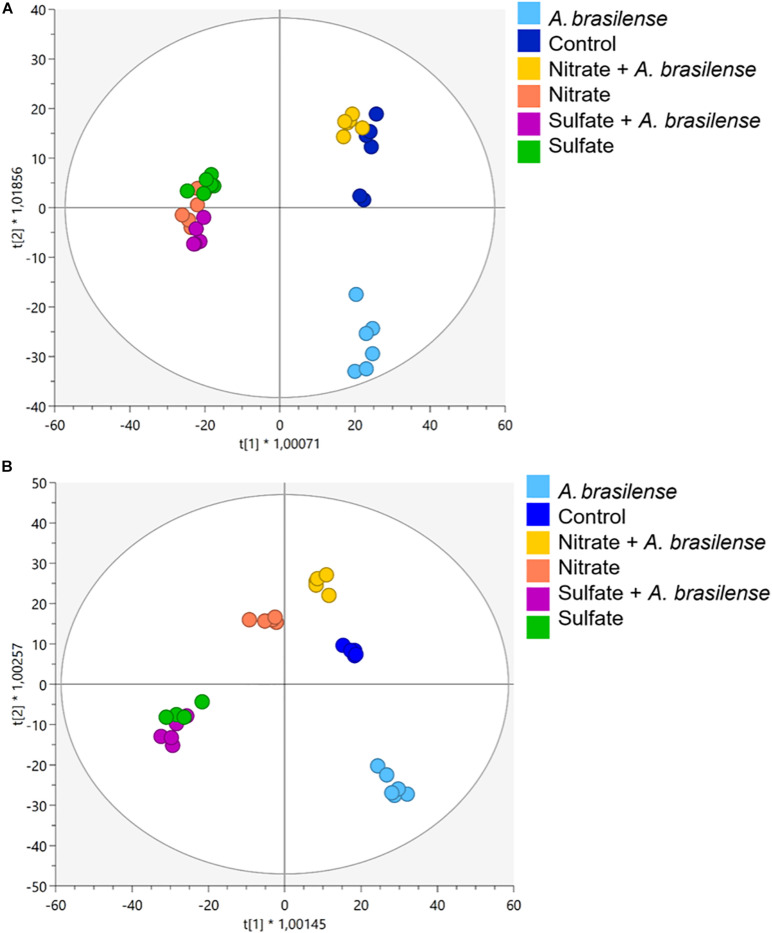
Orthogonal projection to latent structures discriminant analysis (OPLS-DA) supervised modeling of metabolomic signatures in sweet basil plants of cv. **(A)** *Genovese* and **(B)** *Red Rubin*. Plants were grown in control hydroponic solution, in a NO_3_^–^ or in a SO_4_^2–^ overfertilized nutrient solution, either non-inoculated or inoculated with *A. brasilense.* The metabolomic dataset produced through UHPLC-ESI/QTOF-MS was Pareto scaled and then used for the multivariate OPLS-DA modeling.

More than 4,000 compounds were putatively annotated, with secondary metabolism being widely represented. The complete list of annotated metabolites, together with composite mass spectra and relative intensities, are provided as Supplementary Material (Supplementary Table 2 for cv. *Genovese* and Supplementary Table 3 for cv. *Red Rubin*). The multivariate models revealed that changes in nutrient solution and/or inoculation led to a modulation of plant metabolome, as indicated by the unsupervised HCA. In more detail, such modifications resulted from the different nutrient supply (NO_3_^–^ vs. SO_4_^2–^) in both cultivars, and the combination of different fertilizations with bioinoculation (i.e., either with or without *A. brasilense*) (Supplementary Figures 2A,B for cv. *Genovese* and cv. *Red Rubin*, respectively). The further supervised modeling confirmed the separation of the samples in the score space according to treatments (Figure 2). Indeed, OPLS-DA allowed to better separate the different treatment combinations by discriminating predictive and orthogonal components of variance into the score plot hyperspace for both cultivars (Figures 2A,B). The model was validated, and the parameters indicated good predictability both in cv. *Genovese* (R^2^Y = 0.956; Q^2^Y = 0.54; CV-ANOVA, *p* = 3.78E^–4^) and *Red Rubin* (R^2^Y = 0.972; Q^2^Y = 0.55; CV-ANOVA, *p* = 4.72E^–3^). Broadly speaking, OPLS-DA minimized the cultivar-specific behavior as evidenced by HCAs. In fact, the supervised modeling allowed highlighting a common response of both cultivars to the different combined treatment. The first latent vector discriminated control plants and SO_4_^2–^-containing treatments regardless of bioinoculation. Indeed, any consistent separation occurred between the different SO_4_^2–^ treatments (± *A. brasilense*), implying a hierarchically stronger effect of the SO_4_^2–^-based fertilization. On the other hand, for NO_3_^–^ supplementation, some differences can be appreciated considering the two different basil genotypes, suggesting a distinctive metabolic reprogramming at the molecular level. Indeed, *Red Rubin* plants fertilized only with NO_3_^–^ overlapped with SO_4_^2–^-overfertilized samples (± *A. brasilense*), while regarding cv. *Genovese*, the simple overfertilization with NO_3_^–^ generated a separated group, which is closer to the combined treatment NO_3_^–^ + *A. brasilense* and controls. Moreover, plants treated with a combination of NO_3_^–^ and *A. brasilense* appeared to behave likewise with non-inoculated controls in both cultivars. Remarkably, the most distant variance is explained by the simple bioinoculation, which formed a perfectly separated group from all the other treatments.

The metabolites having the highest discrimination potential between treatments were identified by VIP analysis (VIP score > 1.3; Supplementary Table 4). Accordingly, 196 and 267 compounds were identified for *Red Rubin* and *Genovese* cultivars, respectively. About half of the total metabolites related to secondary metabolism, suggesting its strong remodulation in response to the various treatments. The most represented compounds belonged to phenylpropanoids, isoprenoids, and alkaloids. In particular, we underlined the presence of several flavonoids and terpenoids (including carotenoids and brassinosteroids). On the other hand, considering the primary metabolism, the most significant changes are related to fatty acid metabolism and, although to a lesser extent, to carbohydrates and amino acids metabolism.

**FIGURE 3:**
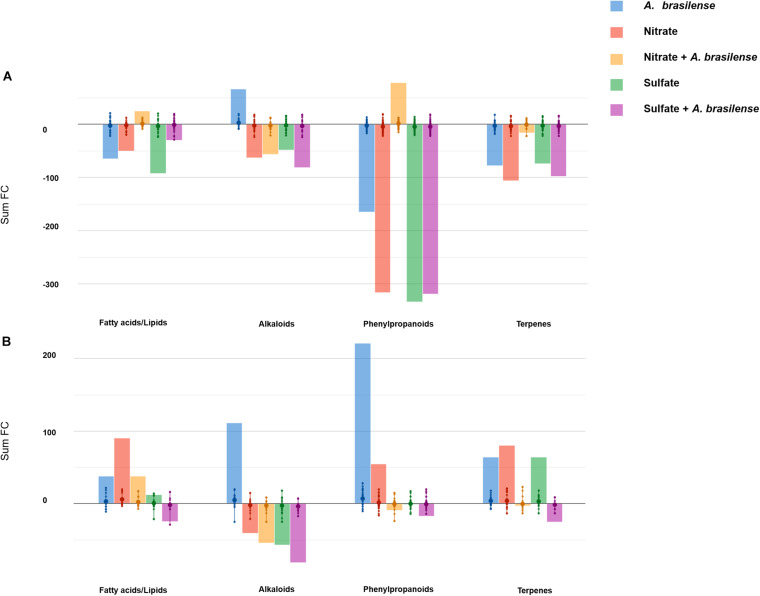
Biosynthesis processes that take place in leaves of sweet basil **(A)** cv. *Genovese* and **(B)** cv. *Red Rubin* plants in response to different treatments. Plants were grown in control hydroponic solution, in a NO_3_^–^- or in a SO_4_^2–^-overfertilized nutrient solution, either non-inoculated or inoculated with *A. brasilense.* The metabolomic dataset produced through UHPLC-ESI/QTOF-MS was subjected to a Volcano Plot analysis (*P* < 0.05, fold-change > 1.5), and differential metabolites were loaded into PlantCyc Pathway Tool (https://www.plantcyc.org/). The *x*-axis represents each set of subcategories, while the *y*-axis corresponds to the cumulative fold-change.

The influence of treatments on the nutritional value of both cultivars was then extrapolated from metabolomic profiles. With this purpose, Volcano analysis was used to identify the leading bioactive compounds differing from control (*p* < 0.05; FC > 1.5; Supplementary Table 5). Differential metabolites were then elaborated using the Pathway Tools Omics Dashboard of PlantCyc, with a data reduction purpose. Figures 3A,B depict the principal classes of health-promoting compounds classified by the Omics Dashboard for *Genovese* and *Red Rubin*, respectively.

The comparison of the two analyses pointed out a striking cultivar-specific response. Indeed cv. *Genovese* had an overall down-accumulation of the secondary metabolism in response to most of the treatments, unlike that observed in *Red Rubin* (that broadly accumulated these classes of compounds). Despite the negative modulation related to S and N supply and/or inoculation in cv. *Genovese*, the treatments elicited specific functional compounds. In this sense, the inoculation with *A. brasilense* enhanced the accumulation of alkaloids, while flavonoids increased in the presence of the combined treatment NO_3_^–^ + *A. brasilense*. This latter treatment elicited the accumulation of high amounts of health-promoting compounds (for instance, the precursor of provitamin A, prephytoene diphosphate, tricetin, and the coumarin scopanone) and promoted the biosynthesis of unsaturated fatty acids.

*A. brasilense* induced the highest effect on secondary metabolites since not only alkaloids were up-accumulated, as in the case of cv. *Genovese*, but also terpenoids and phenylpropanoids were strongly elicited by the microorganism. Several precursors were up-accumulated after inoculation, suggesting a modulation of upstream biosynthetic processes. Regarding NO_3_^–^-overfertilized plants, unsaturated fatty-acid-related compounds were up-accumulated, as well as terpenoids, including diterpenes and triterpenes, while tetraterpenes seemed to be repressed. SO_4_^2–^ and, largely, SO_4_^2–^ + *A. brasilense* presented a negative effect on secondary metabolism and fatty acids in cv. *Red Rubin*.

### Phenolic Acids

**FIGURE 4:**
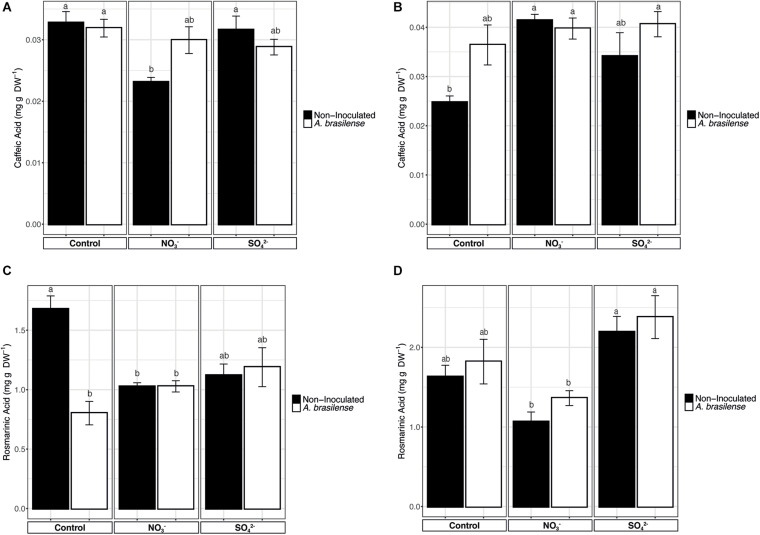
Concentration of phenolic acids**. (A)** Concentration of caffeic acid in sweet basil cv. *Genovese* plants grown in control hydroponic solution, in a NO_3_^–^ or in a SO_4_^2–^ overfertilized nutrient solution, either non-inoculated or inoculated with *A. brasilense*. **(B)** Concentration of caffeic acid in sweet basil cv. *Red Rubin* plants grown in control hydroponic solution, in a NO_3_^–^ or in a SO_4_^2–^ overfertilized nutrient solution, either non-inoculated or inoculated with *A. brasilense*. **(C)** Concentration of rosmarinic acid in sweet basil cv. *Genovese* plants grown in control hydroponic solution, in a NO_3_^–^ or in a SO_4_^2–^ overfertilized nutrient solution, either non-inoculated or inoculated with *A. brasilense*. **(D)** Concentration of rosmarinic acid in sweet basil cv. *Red Rubin* plants grown in control hydroponic solution, in a NO_3_^–^ or in a SO_4_^2–^ overfertilized nutrient solution, either non-inoculated or inoculated with *A. brasilense*. Data are reported as means ± SE, *n* = 6. The statistical significance was tested by means of ANOVA with Tukey posttest. Different letters indicate statistically different values (*p* < 0.05).

A majority of the phenolic compounds present in basil leaves are derivatives of caffeic acid (CA). Therefore, to understand the effects of the mineral nutrients supplementation of the biosynthesis of these secondary metabolites, CA was separated by HPLC and measured along with one of its dimeric derivatives, the rosmarinic acid (RA). The concentration of CA in cv. *Genovese* was not affected by the treatments applied (Figure 4A), except for a slight reduction in the leaves of plants grown in NO_3_^–^ fortified NS. Yet, it is noteworthy that, in the case of cv. *Red Rubin*, the lowest CA concentration was shown in the leaves of the control plants, while the different treatments imposed caused an enhanced accumulation of CA as compared to controls (Figure 4B). However, only the overfertilization with NO_3_^–^ determined a significant increase in CA concentration in both non-inoculated and inoculated plants (Figure 4B), as compared to non-inoculated controls.

The concentration of RA in cv. *Genovese* was significantly influenced by both N and S overfertilization and rhizobacteria (Figure 4C). In particular, the concentration of RA was the highest in control plants, while the overfertilization with NO_3_^–^ and SO_4_^2–^ and the inoculation with *A. brasilense* caused a significant reduction (Figure 4C). On the other hand, in cv. *Red Rubin*, the fertilization practices showed to have opposite effects on RA concentration in leaves (Figure 4D). In fact, plants fertilized with increased concentration of SO_4_^2–^ presented a significantly higher concentration of RA as compared to plants supplemented with NO_3_^–^ (Figure 4D). However, neither the NO_3_^–^ nor the SO_4_^2–^ overfertilization caused a significantly different accumulation of RA as compared to control plants, as well as the inoculation with *A. brasilense* (Figure 4D).

### Amino Acids

The results of the total amino acids analysis are reported in Table 1 and highlighted that, in both *Genovese* and *Red Rubin* cultivars, arginine (Arg) was the most affected by the treatments in terms of relative abundance. In fact, the overfertilization with NO_3_^–^ caused an increase of about 30 times in both cultivars as compared to non-inoculated control, while the supplementation with 8 mM SO_4_^2–^ induced enhancement in the concentration of approximately 50- and 40-fold in cv. *Genovese* and *Red Rubin*, respectively, with respect to control. In the case of NO_3_^–^ fertilized *Genovese* plants, the inoculation with *A. brasilense* caused an additional increase in Arg concentration that was not recorded in SO_4_^2–^-treated plants. Similarly, the inoculation of *Red Rubin* plants did not induce a significant alteration in the Arg concentration of leaves. In addition, in the cv. *Genovese*, the treatments imposed were not affecting the concentration of aspartic acid (Asp), glutamic acid (Glu), phenylalanine (Phe), proline (Pro), and valine (Val), while alanine (Ala), cysteine (Cys), glycine (Gly), leucine (Leu), and threonine (Thr) were decreased by both the fertilization practices and the inoculation with *A. brasilense* as compared to non-inoculated controls. Furthermore, the amino acids lysine (Lys), methionine (Met), and serine (Ser) were significantly increased by the fertilization with SO_4_^2–^, while the supplementation with NO_3_^–^ induced a slight concentration enhancement as compared to control plants, albeit not significantly. In cv. *Red Rubin*, Ala, Asp, Gly, Met, Phe, and Ser were not affected by the treatments, while Cys, Leu, and Thr concentrations were reduced by both the fertilization practices and the inoculation with *A. brasilense* as compared to non-inoculated controls. Nonetheless, in the cv. *Red Rubin*, a group of amino acids composed of Glu, Lys, Phe, Pro, and Val showed an increasing trend in response to the treatments applied with respect to non-inoculated controls.

**TABLE 1:** Amino Acid concentration in both cv. *Genovese* and cv. *Red Rubin* plants grown in control hydroponic solution, in a NO_3_^–^ or in a SO_4_^2–^ overfertilized nutrient solution, either non-inoculated or inoculated with *A. brasilense.*

**Basil cultivar**	**Treatments**	**Ala**		**Arg**		**Asp**		**Cys**		**Glu**		**Gly**		**Leu**		**Lys**		**Met**		**Phe**		**Pro**		**Ser**		**Thr**		**Val**	
		**Mean**	**SE**	**Mean**	**SE**	**Mean**	**SE**	**Mean**	**SE**	**Mean**	**SE**	**Mean**	**SE**	**Mean**	**SE**	**Mean**	**SE**	**Mean**	**SE**	**Mean**	**SE**	**Mean**	**SE**	**Mean**	**SE**	**Mean**	**SE**	**Mean**	**SE**
*Genovese*	Control	3.19^*ns*^	0.59^a^	0.71	0.05^c^	2.06	0.23^*ns*^	697.66	23.50^a^	20.61	2.38^*ns*^	121.76	9.67^a^	2.06	0.11^a^	2.76	1.44^ab^	0.59	0.05^bc^	17.11	4.57^*ns*^	23.76	2.78^*ns*^	12.40	0.81^b^	38.30	6.02^a^	10.69	1.85^*ns*^
	Control + *A. brasilense*	3.27	0.43^a^	1.77	0.19^bc^	2.90	0.26	498.69	30.69^b^	20.65	2.24	146.92	15.90^a^	2.09	0.03^a^	1.64	0.35^b^	0.53	0.01^c^	15.04	3.13	23.47	3.05	18.30	2.32^ab^	50.98	11.84^a^	10.65	1.45
	Nitrate	0.84	0.05^b^	15.57	3.36^bc^	3.56	0.76	71.24	17.15^c^	23.77	1.90	51.88	8.17^c^	0.69	0.04^b^	3.87	0.50^ab^	0.84	0.06^ab^	12.56	2.22	25.51	0.74	18.74	1.81^ab^	7.40	0.91^b^	23.24	6.06
	Nitrate + *A. brasilense*	1.04	0.12^b^	31.94	2.44^abc^	3.00	0.40	60.03	8.00^c^	29.68	3.51	62.46	4.92^c^	0.62	0.03^b^	5.23	1.71^ab^	0.67	0.10^bc^	21.59	7.94	24.19	0.97	33.34	14.30^ab^	5.61	1.39^b^	16.52	3.55
	Sulfate	1.27	0.20^b^	71.26	14.82^a^	10.40	4.95	70.12	10.22^c^	29.62	2.48	106.58	9.07^ab^	0.67	0.05^b^	5.88	0.50^a^	1.06	0.09^a^	21.01	2.02	28.99	1.35	45.73	11.01^a^	6.45	2.09^b^	23.37	7.73
	Sulfate + *A. brasilense*	1.01	0.03^b^	45.31	20.08^ab^	5.88	1.15	64.64	6.76^c^	33.31	8.01	69.20	10.23^bc^	0.68	0.07^b^	4.17	0.28^ab^	1.03	0.05^a^	16.09	2.36	32.99	4.58	27.75	4.07^a^	4.41	1.32^b^	22.15	5.87
*Red Rubin*	Control	2.08	0.27^*ns*^	0.69	0.06^b^	2.06	0.42^*ns*^	502.54	15.75^a^	14.50	2.65^b^	115.21	19.06^*ns*^	2.29	0.06^a^	1.27	0.18^b^	0.59	0.02^*ns*^	9.74	1.33^*ns*^	11.20	1.25^b^	9.61	1.13^*ns*^	45.97	7.96^a^	8.68	1.22^b^
	Control + *A. brasilense*	2.18	0.31	2.32	0.12^b^	2.78	0.32	481.25	34.18^a^	17.91	1.64^b^	173.72	12.19	2.30	0.04^a^	1.14	0.05^b^	0.69	0.12	10.75	1.02	15.53	2.74^b^	12.20	0.88	50.54	8.91^a^	9.87	1.19^b^
	Nitrate	0.91	0.12	37.31	10.71^a^	10.09	5.35	61.64	10.93^b^	19.56	0.99^b^	114.18	43.97	0.70	0.11^bc^	4.42	1.03^a^	0.47	0.08	9.21	0.66	26.43	2.17^a^	10.62	1.04	8.97	5.74^b^	22.61	6.59^a^
	Nitrate + *A. brasilense*	1.42	0.33	22.78	8.29^ab^	2.81	0.18	56.81	0.83^b^	21.49	1.57^ab^	118.24	23.79	1.24	0.28^b^	2.30	0.44^ab^	0.72	0.09	9.99	1.20	14.61	2.87^b^	12.38	1.43	16.87	8.18^b^	13.25	1.24^ab^
	Sulfate	3.07	1.51	38.79	9.17^a^	6.16	1.49	43.05	3.32^b^	30.19	3.85^a^	83.65	16.92	0.51	0.02^c^	3.21	0.39^ab^	0.70	0.01	9.39	1.20	29.71	1.10^a^	16.02	3.36	3.24	0.45^b^	13.49	1.07^ab^
	Sulfate + *A. brasilense*	1.21	0.04	42.30	5.69^a^	3.74	0.21	43.43	1.31^b^	22.69	1.74^ab^	89.16	9.04	0.60	0.08^c^	3.41	0.96^ab^	0.61	0.03	11.94	2.42	30.81	0.20^a^	13.67	1.48	2.69	0.41^b^	10.82	0.55^ab^

*The concentration is expressed as mmol g DW^–1^, and the data are reported means ± SE, n = 6. The statistical significance was tested by means of ANOVA with Tukey posttest. Different letters indicate statistically different values (p < 0.05).*

## Mineral Nutrients Content

### Ionomic Analysis

**FIGURE 5:**
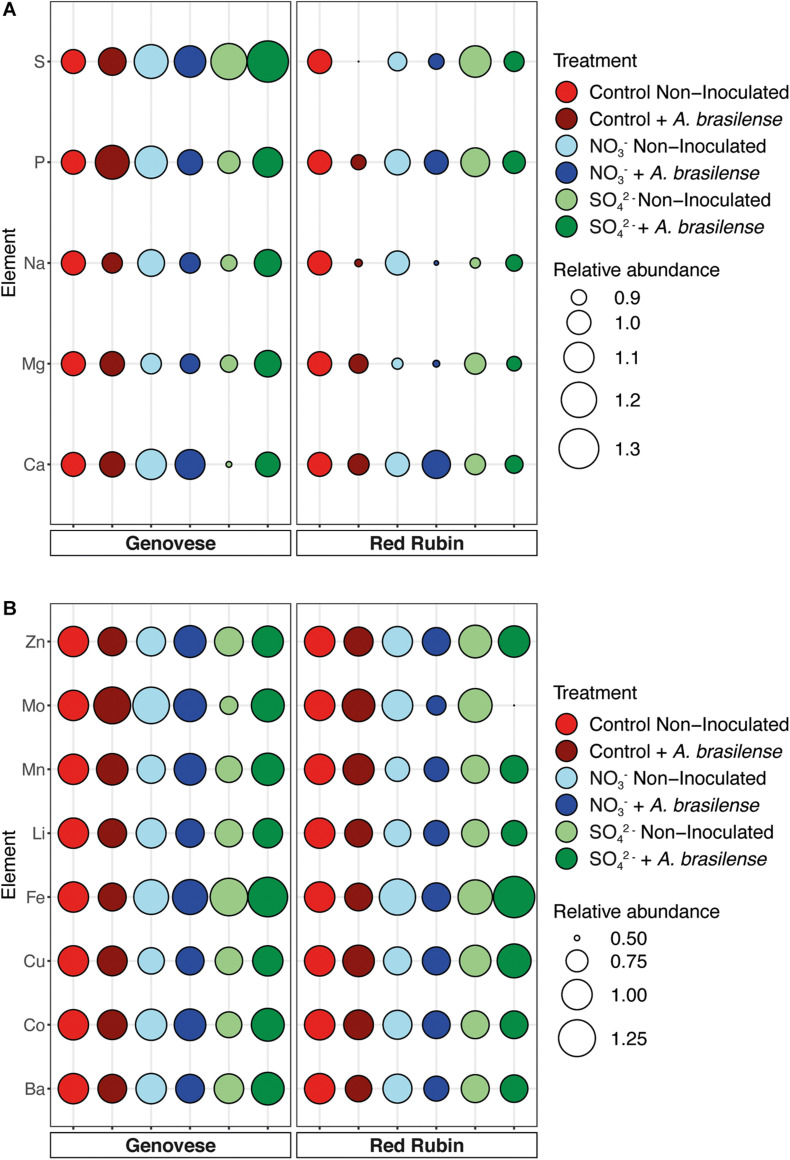
Ionomic profile of basil plants. **(A)** Relative abundance of macronutrients in both cv. *Genovese* and cv. *Red Rubin* plants grown in control hydroponic solution, in a NO_3_^–^- or in a SO_4_^2–^-overfertilized nutrient solution, either non-inoculated or inoculated with *A. brasilense*. **(B)** Relative abundance of micronutrients in both cv. *Genovese* and cv. *Red Rubin* plants grown in control hydroponic solution, in a NO_3_^–^- or in a SO_4_^2^-overfertilized nutrient solution, either non-inoculated or inoculated with *A. brasilense*. Relative abundance has been calculated by normalizing the concentration of each single element in all the different treatments considered to the concentration of the fame element in the non-inoculated control sample. Concentration data and statistical analyses are reported in Supplementary Tables 6,7.

To understand whether the different treatments might have influenced the uptake and allocation of mineral nutrients, the whole ionome profile of basil leaves, at the end of the cultivation period, was analyzed through ICP-OES. In the case of cv. *Genovese*, the macronutrients were mostly unaffected by the treatments imposed, except for calcium (Ca) and S (Figure 5A and Supplementary Table 6). Indeed, the treatment with NO_3_^–^ induced the highest accumulation of Ca in the leaves of basil, independently from the inoculation with *A. brasilense*. As expected, the highest concentration of S was detected in the leaves of plants grown in SO_4_^2–^-fortified NS, with *Azospirillum* showing a further promoting effect on S accumulation at leaf level (Figure 5A and Supplementary Table 6). Concerning the micronutrients concentration in cv. *Genovese* plants, the only remarkable effects was shown by iron (Fe). In fact, both NO_3_^–^ and SO_4_^2–^ overfertilization had a promoting effect on Fe accumulation at leaf level. Interestingly, the highest Fe concentration was shown by basil plants fertilized with 8 mM SO_4_^2–^ and inoculated with *A. brasilense* (Figure 5B and Supplementary Table 7). Plants of *Red Rubin* cultivar showed a decreasing trend in the accumulation of the macronutrient magnesium (Mg) in the leaves upon treatment. This reduction was statistically significant in NO_3_^–^ fertilized plants, independently of the inoculation with *A. brasilense*, as compared to non-inoculated control plants (Figure 5A and Supplementary Table 6). As also observed for cv. *Genovese*, the fertilization with SO_4_^2–^ caused the highest accumulation of S in the leaves of cv. *Red Rubin* plants. These latter also displayed an increased concentration of phosphorus (P) as compared to the other samples (Figure 5A and Supplementary Table 6). The fertilization with NO_3_^–^ caused in *Red Rubin* cultivar a significant decrease in Cu concentration in leaves, while the combination of SO_4_^2–^ fertilization and the inoculation with *A. brasilense* induced the opposite effect, determining the highest accumulation of the micronutrient Cu (Figure 5B and Supplementary Table 7). In the control *Red Rubin* plants, the inoculation with *A. brasilense* caused a slight increase in the concentration of both manganese (Mn) and molybdenum (Mo), albeit not significantly. In contrast, the fertilization treatments induced a decreasing trend as compared to the control plants (Figure 5B and Supplementary Table 7).

### Nitrate Content

**FIGURE 6:**
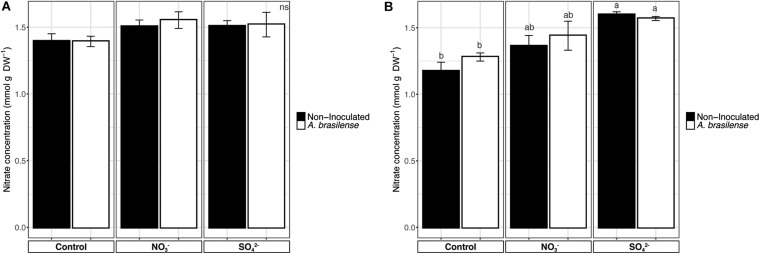
Nitrate concentration in basil leaves. **(A)** Nitrate concentration in sweet basil cv. *Genovese* plants grown in control hydroponic solution, in a NO_3_^–^- or in a SO_4_^2–^-overfertilized nutrient solution, either non-inoculated or inoculated with *A. brasilense*. **(B)** Nitrate concentration in sweet basil cv. *Red Rubin* plants grown in control hydroponic solution, in a NO_3_^–^- or in a SO_4_^2–^-overfertilized nutrient solution, either non-inoculated or inoculated with *A. brasilense*. Data are reported as means ± SE, *n* = 6. The statistical significance was tested by means of ANOVA with Tukey posttest. Different letters indicate statistically different values (*p* < 0.05).

The analyses of nitrate in sweet basil leaves showed that, in cv. *Genovese*, the NO_3_^–^ concentration was comprised between 1.39 and 1.5 mmol g^–1^ DW and that it was unaffected by the treatments imposed (Figure 6A). On the other hand, cv. *Red Rubin* displayed an increase in NO_3_^–^ concentration in the leaves according to the overfertilization practices, reaching values of 1.3 and 1.6 mmol g^–1^ DW in NO_3_^–^- and SO_4_^2–^-treated plants, respectively (Figure 6B).

## Discussion

Among the mineral elements, nitrogen (N) and sulfur (S) are accounted as essential macronutrients for plants, significantly determining the yield and quality of crops (Marschner, 2012). Increased growth and development in plants fertilized with higher concentrations of macronutrients or inoculated with PGPR have been reported for several species (Egamberdieva and Teixeira da Silva, 2015; Orhan et al., 2006; Fan et al., 2017). The data obtained displayed that the effects of nitrogen and sulfur fertilization on sweet basil are consistent with previous reports with fresh biomass showing an increasing trend as determined by micronutrients supplementation (Zheljazkov et al., 2008; Kiferle et al., 2011; Oliveira et al., 2014). Indeed, N fertilization has already been shown to be one of the pivotal factors affecting basil yield (Zheljazkov et al., 2008). Similarly, treatments with S also increased plant yield, especially in the case of cv. *Genovese*. Although Oliveira et al. (2014) reported that S fertilizer increased biomass production only at the root level, in the present study, shoot biomass of cv. *Genovese* is also increased. *Red Rubin* cultivar, on the other hand, was not affected by S treatments, possibly highlighting a different response of the two cultivars toward the fertilization practices.

Despite the promoting effects observed brought about by specific *A. brasilense* strains on basil growth (Mangmang et al., 2016), the inoculation did not induce significant alteration in the biomass of cv. *Genovese*, while it produced a higher root development in cv. *Red Rubin*. Up to now, *Azospirillum* inoculation has been shown to induce various effects on plant physiological parameters; in fact, it has been demonstrated to increase plant growth parameters in strawberry (Guerrero-Molina et al., 2014; Pii et al., 2018) and corn (Zaady et al., 1993; Pii et al., 2019). Nonetheless, it has also been demonstrated that the growth-promoting effects of *A. brasilense* are strongly dependent on plant species and genotypes (Pedraza et al., 2010; Pii et al., 2018). In the specific case of basil plants, Raei et al. (2015) revealed that *Azospirillum* inoculation improved plant growth parameters and resistance to drought stress, while Roshanpour et al. (2014) reported how the combination of three different rhizobacteria (*Azotobacter*, *Azospirillum*, and *Bacillus*) positively influenced the fresh and dry yield of basil, as the essential oil yield. Besides, recent evidence has also highlighted that *A. brasilense* affects the nutraceutical profile of hydroponically grown strawberry plants (Pii et al., 2018), induces secondary metabolism in oregano (Erika et al., 2010), and alters the root exudation profiles in cucumber (Pii et al., 2015c), thus suggesting a direct influence on plants metabolome.

Plant metabolomics emerges as a powerful approach to broaden knowledge about the biochemical profile of plant-based foods, giving the possibility to control and improve their nutritional value and to establish targeted strategies (Hall et al., 2008). In our study, the untargeted metabolomics analysis unraveled a distinct metabolic reprogramming of the two cultivars (i.e., *Genovese* and *Red Rubin*) of sweet basil in response to the different combinations of mineral supplementation (S/N) and bioinoculation with *A. brasilense*. In fact, the two cultivars showed peculiar metabolic responses, confirming the primary influence of the genetic background on the production of bioactive compounds in hydroponically grown basil plants, as previously reported (Salas-Pérez et al., 2018). These results pointed out the pivotal role of the selected genotype in shaping the profile of health-promoting compounds in basil.

Besides the genetic background, several other factors could alter the basil composition, such as the growing conditions and agronomic practices (Corrado et al., 2020). Notably, the nutritional value of plant-based food is usually correlated with the accumulation of secondary metabolites, which are largely modulated by environmental conditions (Rouphael and Kyriacou, 2018). In fact, secondary metabolites represent an important part of the human diet. For instance, phenolic compounds are considered as powerful antioxidants protecting against oxidative damage (Lin et al., 2016). Likewise, plant terpenes include essential vitamers for humans as well as important health-promoting compounds such as squalene or carotenoids (Tetali, 2019). Similarly, a large amount of alkaloids present antimicrobial, antihypertensive, and antineuroinflammatory activity, and some of them, in particular vinblastine and vincristine, have proved to be anticancer compounds (Almagro et al., 2015). Among the functional compounds found in basil leaves, terpenoids and phenylpropanoids have been reported among the most accumulated compounds (Rouphael and Kyriacou, 2018). Although it is known that resource-limited environments promote secondary metabolism, the carbon flux between primary and secondary pathways seems to be more complex (Fritz et al., 2006). Nitrogen is required not only in carbon metabolism for the essential physiological processes but also in the biosynthesis of precursors for the secondary metabolism (Fritz et al., 2006). Similarly, S is the precursor of many chemoprotective compounds and takes part in plant processes. Carbon-based secondary metabolites are postulated to be inversely correlated with nitrogen availability, and nitrogen-based secondary metabolites directly correlated (Heimler et al., 2017). In our study, S- and N-based fertilizer induce repression of secondary metabolism in *Genovese* cultivar. Surprisingly, both *Genovese* and *Red Rubin* cultivars presented a general down-accumulation of alkaloids, suggesting that the plant might promote either growth or differentiation under resource-rich environments, according to the growth/differentiation balance hypothesis (Rembialkowska, 2007). However, a complex alteration regarding carbon-based secondary metabolites took place in the *Red Rubin*-treated plants since phenylpropanoids and terpenoids were mostly accumulated. Although many authors revealed that the N deprivation enhanced the phenylpropanoids biosynthesis as in the case of *Red Rubin* cultivar, Heimler et al. (2017) pointed out that inorganic nutrition diversely modulates the specific classes of phenolics and, according to our results, depends on the genotype. The down-accumulation observed in the treated plants of cv. *Genovese* agreed with previous studies summarized by Albornoz (2016), who revealed that N overfertilization would imply a loss of crop quality by decreasing bioactive compounds as ascorbic acid or phenylpropanoids (Albornoz, 2016). Consistently with the metabolomic analyses, the targeted quantification of caffeic acid did not reveal any specific alteration in the leaves’ accumulation pattern as affected by the treatment imposed, whereas rosmarinic acid (RA) was significantly down-accumulated in cv. *Genovese* plants. Nonetheless, according to Kiferle et al. (2011), the accumulation of RA in basil plants is maximized at flowering stage; therefore, it is not surprising that the levels detected in our analyses are lower than the concentrations found in previous pieces of research (Kiferle et al., 2011). Interestingly, the treatments imposed also caused the significative accumulation of the amino acid Arg, which, besides being essential for the biosynthesis of proteins, also plays a pivotal role as a precursor of multiple secondary metabolites, polyamines, and nitric oxide. In addition, Arg can be frequently used by plants as major nitrogen storage form in seeds and other vegetative tissues. Its mobilization can indeed provide a readily usable N flux for different physiological processes (Todd and Gifford, 2002; Cánovas et al., 2007; Babst and Coleman, 2018).

On the other hand, *Azospirillum* spp. might increase plant quality not only by assimilating atmospheric inorganic N but also by triggering signaling molecules. In fact, *Azospirillum* spp. have been reported to synthesize phytohormones, including auxins, cytokinins, and gibberellins. Interestingly, *Azospirillum* also produces stress-related molecules like jasmonic acid, abscisic acid, ethylene, and nitric oxide (Fukami et al., 2018). This complex network of key molecules in plant response could explain the modulation of secondary metabolism we observed. The distinct response based on the genotypes has been previously reported (Sasaki et al., 2010; Chamam et al., 2013). Moreover, the addition of inorganic elements (N/S) also modified the plant response to the microorganism as reported by Sasaki et al. (2010), who observed that the plant response to *Azospirillum* inoculation depended on the N level.

It has been widely assessed that the growth conditions (i.e., chemical and physical characteristics of the growth substrate, fertilization practices, and inoculation with PGPR) can affect the mineral composition of agricultural plants, sometimes producing an increase in the concentration of either essential or non-essential mineral elements with health-promoting effects for consumers (Tomasi et al., 2009; Pii et al., 2015a; Astolfi et al., 2018). Interestingly, the treatment with NO_3_^–^, independently of the presence of *A. brasilense*, caused the accumulation of Ca in the leaves of cv. *Genovese*. Calcium plays an essential role as macronutrient for plants and animals, and it has paramount importance from a structural and biochemical (i.e., signaling) point of view. Being that major staple crops are poor sources of Ca, it has been observed that low dietary intake of this macronutrients in humans is epidemiologically linked to various diseases, which can have serious health consequences over time (Sharma et al., 2017). As expected, the fertilization with SO_4_^2–^ determined a significant increase in the concentration of S in both cultivars. Indeed, the importance of S as health-promoting elements is represented by the fact that it is contained in the chemical structure of bioactive phytochemicals, for instance, related to the scavenging of oxidative stress (González-Morales et al., 2017). As previously demonstrated, the increased S fertilization is connected with an enhanced ability of plants to take up and store Fe (Astolfi et al., 2006, 2018; Zuchi et al., 2012; Celletti et al., 2016), whose concentration resulted increased in both *Genovese* and *Red Rubin* cultivars. Consistently, as previously observed (Nikolic et al., 2007), NO_3_^–^ provision is a fundamental prerequisite for an efficient reduction and uptake of Fe in dicot plants, thus leading to an increased microelement concentration in the leaves of both basil cultivars. Yet, the inoculation with *A. brasilense*, which was shown to be effective in enhancing the Fe content in cucumber and maize plants (Pii et al., 2015c, 2016, 2019), did not display any influence in the Fe uptake and allocation in basil plants, further underlining the species-specific response of plants to the inoculation with the PGPR. On the other hand, the SO_4_^2–^ fertilization and the inoculation with *A. brasilense* caused the increase in the concentration of Cu in cv. *Red Rubin*, which can result in toxicity for plants above a certain threshold and even an antinutritional element for human consumption (Brunetto et al., 2016). However, according to the European Commission directives^3^, the Cu concentrations detected in the basil leaves in our experimental model are not considered harmful to human health. Similarly, the fertilization with N sources might lead to the accumulation of NO_3_^–^ in the edible parts of the plants, which is demonstrated to have negative effects for consumers (Santamaria, 2006). Nonetheless, despite the increase in NO_3_^–^ observed, especially in cv. *Red Rubin*, the concentrations detected cannot be considered toxic, since they are lower than those reported in the literature (Chang et al., 2013).

In conclusion, our results underline that genotype was the main factor in differentiating plant response to the treatments in terms of biomass production and nutraceutical compounds accumulation. As an example, the increase in the conditionally essential amino acid Arg and the essential amino acid Met, as well as the elicitation of carotenoids and phenolics like phenylpropanoids and phenolic acids, worth to be considered. Even though both genotypes modulate the metabolism of unsaturated fatty acids, flavonoids, alkaloids, and several terpene derivatives, which are well-known compounds for their role in the human health and nutrition, the *Red Rubin* cultivar showed the most positive effect in terms of nutritional value. This impact was more remarkable after *A. brasilense* inoculation. Regardless of the genotype considered, the possibility to modulate the profile of functional compounds, as well as to elicit the accumulation of essential mineral nutrients in the leaves of basil plants, offers promising perspectives concerning the functional role of basil and, possibly, of related herb crops. Nonetheless, it is important to consider that basil response was strongly dependent on the specific treatment considered in a cultivar-dependent manner, rather than exhibiting a generalized modulation of the phytochemical profile.

## Data Availability Statement

The original contributions presented in the study are included in the article/Supplementary Material, further inquiries can be directed to the corresponding author.

## Author Contributions

SC and YP designed the study. SK, MM, LL, BM-M, VB, FV, and YP performed the experiments. SC, YP, MT, TM, LL, BM-M, VB, and SK analyzed and discussed the data. YP, LL, TM, SC, BM-M, and VB wrote the manuscript. All authors contributed to the article and approved the submitted version.

## Conflict of Interest

The authors declare that the research was conducted in the absence of any commercial or financial relationships that could be construed as a potential conflict of interest.

## Footnotes

1 http://www.plantcyc.org

2 http://cosmos-fp7.eu/msi

3 https://ec.europa.eu/food/sites/food/files/safety/docs/sci-com_scf_out176_en.pdf

## Supplementary Material

Supplementary Figure 1 SPAD index of basil plants. (**A).** SPAD index of cv. *Genovese* plants grown in control hydroponic solution, in a NO_3_^–^ or in a SO_4_^2–^ over-fertilized nutrient solution, either non-inoculated or inoculated with *A. brasilense*. (**B).** SPAD index of cv. *Red Rubin* plants grown in control hydroponic solution, in a NO_3_^–^ or in a SO_4_^2–^ over-fertilized nutrient solution, either non-inoculated or inoculated with *A. brasilense*. Data are reported as means ± SE, *n* = 6.

Supplementary Figure 2 Unsupervised hierarchical cluster analysis carried out from UHPLC-ESI/QTOF-MS metabolomic analysis of sweet basil leaves of cv. *Genovese* **(A)** and cv. *Red Rubin* **(B)** in response to different treatments. Plants were grown in control hydroponic solution, in a NO_3_^–^ or in a SO_4_^2–^ over-fertilized nutrient solution, either non-inoculated (−) or inoculated (+) with *A. brasilense*. The fold-change based heat map was used to build hierarchical clusters (linkage rule: Ward; distance: Euclidean).

Supplementary Table 1 Composition of the modified nutrient solutions.

Supplementary Table 2 Whole dataset produced from untargeted metabolomic analysis carried out in leaves of sweet basil leaves of cv. *Genovese* in response to different treatments. Plants were grown in control hydroponic solution, in a NO_3_^–^ or in a SO_4_^2–^ over-fertilized nutrient solution, either non-inoculated or inoculated with *A. brasilense*.

Supplementary Table 3 Whole dataset produced from untargeted metabolomic analysis carried out in leaves of sweet basil leaves of cv. *Red Rubin* in response to different treatments. Plants were grown in control hydroponic solution, in a NO_3_^–^ or in a SO_4_^2–^ over-fertilized nutrient solution, either non-inoculated or inoculated with *A. brasilense*.

Supplementary Table 4 VIP discriminant compounds identified as discriminant from OPLS-DA in leaves of sweet basil plants. Plants were grown in control hydroponic solution, in a NO_3_^–^ or in a SO_4_^2–^ over-fertilized nutrient solution, either non-inoculated or inoculated with *A. brasilense*.

Supplementary Table 5 Differential metabolites as derived from Volcano analysis (*P* < 0.05; FC > 1.5) of leaves of sweet basil plants. Plants were grown in control hydroponic solution, in a NO_3_^–^ or in a SO_4_^2–^ over-fertilized nutrient solution, either non-inoculated or inoculated with *A. brasilense*.

Supplementary Table 6 Macronutrients concentration in both cv. *Genovese* and cv. *Red Rubin* plants grown in control hydroponic solution, in a NO_3_^–^ or in a SO_4_^2–^ over-fertilized nutrient solution, either non-inoculated or inoculated with *A. brasilense*. The concentration is expressed as mg gDW^–1^ and the data are reported means ± SE, *n* = 3. The statistical significance was tested by means of ANOVA with Tukey post-test. Different letters indicate statistically different values (*p* < 0.05).

Supplementary Table 7 Micronutrients concentration in both cv. *Genovese* and cv. *Red Rubin* plants grown in control hydroponic solution, in a NO_3_^–^ or in a SO_4_^2–^ over-fertilized nutrient solution, either non-inoculated or inoculated with *A. brasilense*. The concentration is expressed as μg gDW^–1^ and the data are reported means ± SE, *n* = 3. The statistical significance was tested by means of ANOVA with Tukey post-test. Different letters indicate statistically different values (*p* < 0.05).
