# Supplementary figures and images for: Nutraceutical Profiles of Two Hydroponically Grown Sweet Basil Cultivars as Affected by the Composition of the Nutrient Solution and the Inoculation With Azospirillum brasilense
Source: Front Plant Sci. 2020 Nov 5;11:596000. doi: 10.3389/fpls.2020.596000 (PMC7674207; doi:10.3389/fpls.2020.596000)

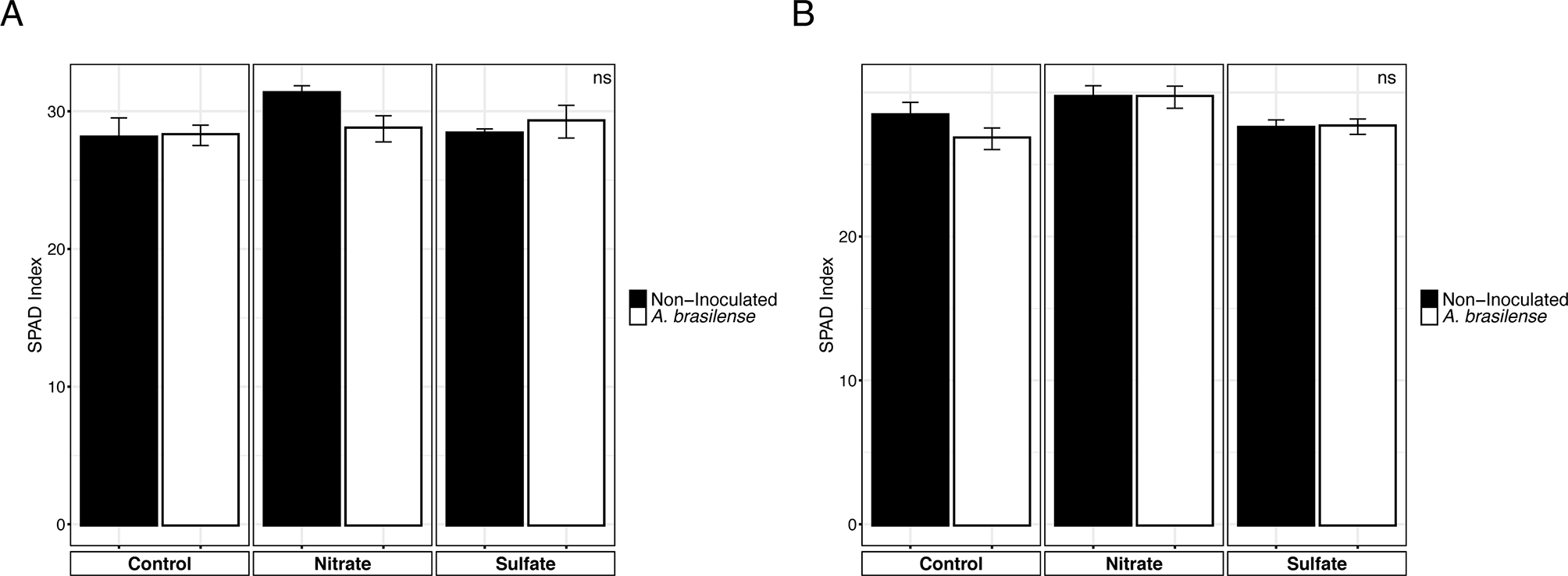

Supplement: Supplementary Figure 1 — SPAD index of basil plants. (A). SPAD index of cv. Genovese plants grown in control hydroponic solution, in a NO3– or in a SO42– over-fertilized nutrient solution, either non-inoculated or inoculated with A. brasilense. (B). SPAD index of cv. Red Rubin plants grown in control hydroponic solution, in a NO3– or in a SO42– over-fertilized nutrient solution, either non-inoculated or inoculated with A. brasilense. Data are reported as means ± SE, n = 6. [file Image_1.TIF]

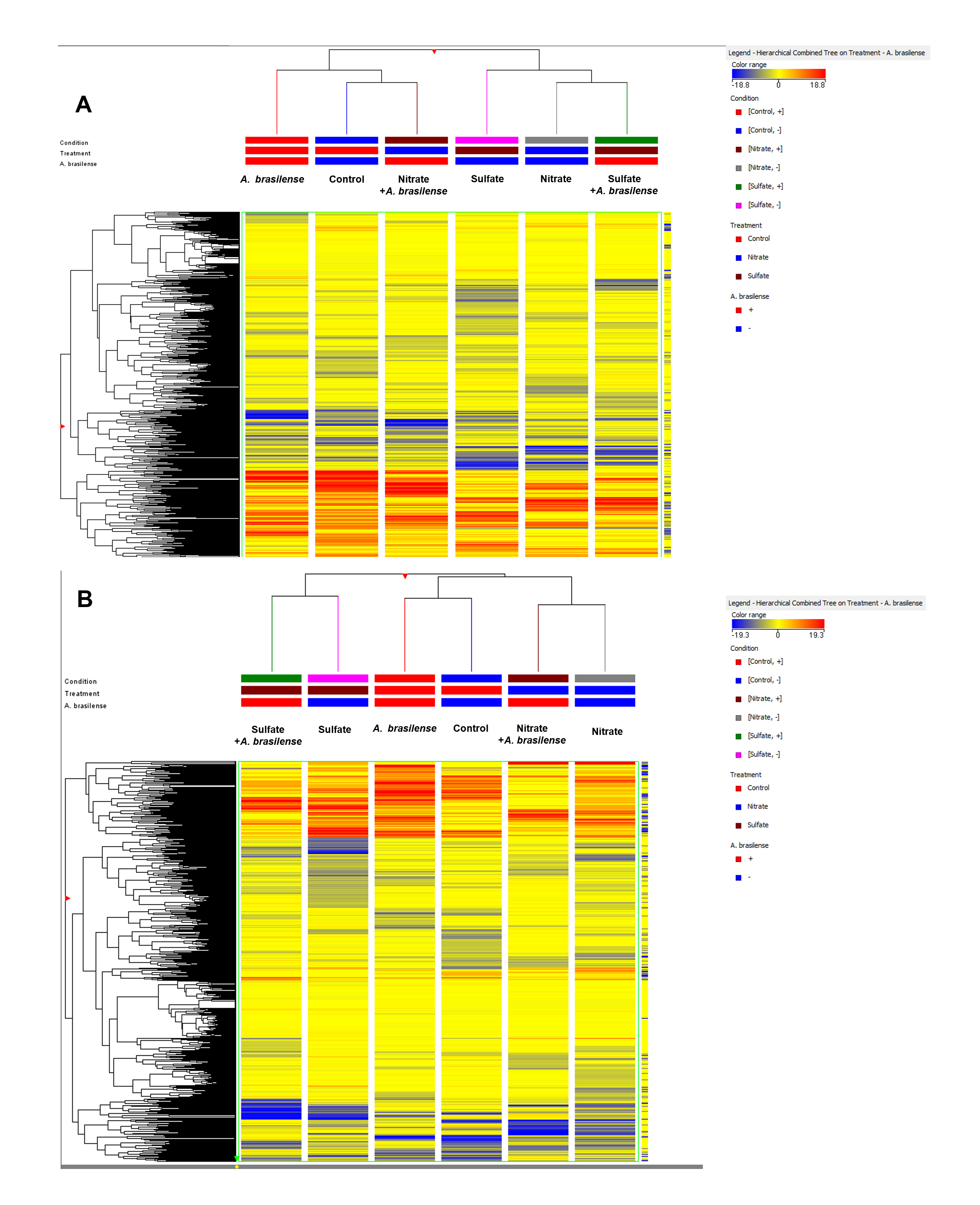

Supplement: Supplementary Figure 2 — Unsupervised hierarchical cluster analysis carried out from UHPLC-ESI/QTOF-MS metabolomic analysis of sweet basil leaves of cv. Genovese (A) and cv. Red Rubin (B) in response to different treatments. Plants were grown in control hydroponic solution, in a NO3– or in a SO42– over-fertilized nutrient solution, either non-inoculated (−) or inoculated (+) with A. brasilense. The fold-change based heat map was used to build hierarchical clusters (linkage rule: Ward; distance: Euclidean). [file Image_2.TIF]
