# Supplementary material for: Nutraceutical Profiles of Two Hydroponically Grown Sweet Basil Cultivars as Affected by the Composition of the Nutrient Solution and the Inoculation With Azospirillum brasilense
Source: Front Plant Sci. 2020 Nov 5;11:596000. doi: 10.3389/fpls.2020.596000 (PMC7674207; doi:10.3389/fpls.2020.596000)
Supplement: Supplementary Table 1 — Composition of the modified nutrient solutions. [file Table_1.DOCX]

**Supplementary Table 1. Composition of the modified nutrient solutions.**

|  | **NO_3_^-^-fortified solution** | **SO_4_^2-^-fortified solution** |
| --- | --- | --- |
| **Macronutrient** | **Concentration (mM)** | **Concentration (mM)** |
| NO_3_^-^ | 20 | 14.5 |
| NH_4_^+^ | 3.2 | 3.8 |
| PO_4_^3-^ | 3.4 | 3.1 |
| K^+^ | 11.2 | 11.6 |
| Mg^2+^ | 3.5 | 4.2 |
| Ca^2+^ | 4.9 | 5 |
| SO_4_^2-^ | 3.1 | 8 |
