# Supplementary material for: Nutraceutical Profiles of Two Hydroponically Grown Sweet Basil Cultivars as Affected by the Composition of the Nutrient Solution and the Inoculation With Azospirillum brasilense
Source: Front Plant Sci. 2020 Nov 5;11:596000. doi: 10.3389/fpls.2020.596000 (PMC7674207; doi:10.3389/fpls.2020.596000)
Supplement: Supplementary Table 6 — Macronutrients concentration in both cv. Genovese and cv. Red Rubin plants grown in control hydroponic solution, in a NO3– or in a SO42– over-fertilized nutrient solution, either non-inoculated or inoculated with A. brasilense. The concentration is expressed as mg gDW–1 and the data are reported means ± SE, n = 3. The statistical significance was tested by means of ANOVA with Tukey post-test. Different letters indicate statistically different values (p < 0.05). [file Table_6.DOCX]

**Supplementary Table 6.** Macronutrients concentration in both cv. Genovese and cv red Rubin plants grown in control hydroponic solution, in a NO_3_^-^ or in a SO_4_^2-^ over-fertilized nutrient solution, either non-inoculated or inoculated with *A. brasilense*. The concentration is expressed as mg gDW^-1^ and the data are reported means ± SE, n = 3. The statistical significance was tested by means of ANOVA with Tukey post-test. Different letters indicate statistically different values (P < 0.05).

| **Basil Cultivar** | **Treatments** | **Ca** | |  | **Mg** | |  | **Na** | |  | **P** | |  | **S** | |  |
| --- | --- | --- | --- | --- | --- | --- | --- | --- | --- | --- | --- | --- | --- | --- | --- | --- |
|  |  | Mean | SE |  | Mean | SE |  | Mean | SE |  | Mean | SE |  | Mean | SE |  |
| **Genovese** | Control | 12.50 | 0.16 | *ab* | 3.95 | 0.08 |  | 1.25 | 0.03 |  | 8.30 | 0.14 |  | 2.57 | 0.15 | *b* |
|  | Control + *A. brasilense* | 12.71 | 0.48 | *ab* | 3.97 | 0.16 |  | 1.19 | 0.01 |  | 9.71 | 0.78 |  | 2.71 | 0.03 | *ab* |
|  | Nitrate | 13.80 | 1.05 | *a* | 3.77 | 0.08 |  | 1.31 | 0.08 |  | 9.46 | 0.48 |  | 3.00 | 0.29 | *ab* |
|  | Nitrate + *A. brasilense* | 13.67 | 1.00 | *a* | 3.72 | 0.15 |  | 1.19 | 0.02 |  | 8.43 | 0.39 |  | 2.88 | 0.05 | *ab* |
|  | Sulfate | 10.55 | 0.29 | *b* | 3.62 | 0.01 |  | 1.14 | 0.00 |  | 8.11 | 0.07 |  | 3.12 | 0.03 | *ab* |
|  | Sulfate + *A. brasilense* | 12.62 | 0.25 | *ab* | 4.10 | 0.01 |  | 1.31 | 0.00 |  | 9.06 | 0.35 |  | 3.43 | 0.20 | *a* |
|  |  |  |  |  |  |  |  |  |  |  |  |  |  |  |  |  |
| **Red Rubin** | Control | 9.92 | 0.05 | *ab* | 3.43 | 0.00 | *a* | 1.32 | 0.03 | *a* | 8.66 | 0.24 | *ab* | 2.58 | 0.07 | *ab* |
|  | Control + *A. brasilense* | 9.53 | 0.08 | *ab* | 3.22 | 0.04 | *ab* | 1.12 | 0.00 | *b* | 7.77 | 0.23 | *b* | 2.16 | 0.09 | *b* |
|  | Nitrate | 9.91 | 0.38 | *ab* | 2.98 | 0.01 | *b* | 1.32 | 0.04 | *a* | 8.83 | 0.14 | *ab* | 2.41 | 0.05 | *ab* |
|  | Nitrate + *A. brasilense* | 10.56 | 0.16 | *a* | 2.91 | 0.08 | *b* | 1.11 | 0.03 | *b* | 8.64 | 0.39 | *ab* | 2.33 | 0.06 | *b* |
|  | Sulfate | 9.48 | 0.33 | *b* | 3.30 | 0.19 | *ab* | 1.14 | 0.00 | *b* | 9.31 | 0.28 | *a* | 2.90 | 0.22 | *a* |
|  | Sulfate + *A. brasilense* | 9.17 | 0.01 | *b* | 3.07 | 0.04 | *ab* | 1.20 | 0.01 | *b* | 8.47 | 0.20 | *ab* | 2.45 | 0.11 | *ab* |
