# Supplementary material for: Nutraceutical Profiles of Two Hydroponically Grown Sweet Basil Cultivars as Affected by the Composition of the Nutrient Solution and the Inoculation With Azospirillum brasilense
Source: Front Plant Sci. 2020 Nov 5;11:596000. doi: 10.3389/fpls.2020.596000 (PMC7674207; doi:10.3389/fpls.2020.596000)
Supplement: Supplementary Table 7 — Micronutrients concentration in both cv. Genovese and cv. Red Rubin plants grown in control hydroponic solution, in a NO3– or in a SO42– over-fertilized nutrient solution, either non-inoculated or inoculated with A. brasilense. The concentration is expressed as μg gDW–1 and the data are reported means ± SE, n = 3. The statistical significance was tested by means of ANOVA with Tukey post-test. Different letters indicate statistically different values (p < 0.05). [file Table_7.DOCX]

**Supplementary Table 7.** Micronutrients concentration in both cv. Genovese and cv red Rubin plants grown in control hydroponic solution, in a NO_3_^-^ or in a SO_4_^2-^ over-fertilized nutrient solution, either non-inoculated or inoculated with *A. brasilense*. The concentration is expressed as µg gDW^-1^ and the data are reported means ± SE, n = 3. The statistical significance was tested by means of ANOVA with Tukey post-test. Different letters indicate statistically different values (P < 0.05).

| **Basil Cultivar** | **Treatments** | **Ba** | |  | **Co** | |  | **Cu** | |  | **Fe** | |  | **Li** | |  | **Mn** | |  | **Mo** | |  | **Zn** | |
| --- | --- | --- | --- | --- | --- | --- | --- | --- | --- | --- | --- | --- | --- | --- | --- | --- | --- | --- | --- | --- | --- | --- | --- | --- |
|  |  | Mean | SE |  | Mean | SE |  | Mean | SE |  | Mean | SE |  | Mean | SE |  | Mean | SE |  | Mean | SE |  | Mean | SE |
| **Genovese** | Control | 1.17 | 0.02 | *ab* | 0.15 | 0.01 | *ab* | 13.81 | 1.49 |  | 138.64 | 8.11 | *c* | 0.57 | 0.01 |  | 107.99 | 8.00 |  | 0.34 | 0.02 |  | 62.44 | 7.55 |
|  | Control + A. brasilense | 1.10 | 0.02 | *b* | 0.14 | 0.00 | *ab* | 13.55 | 0.31 |  | 127.19 | 2.09 | *bc* | 0.54 | 0.01 |  | 112.02 | 10.93 |  | 0.42 | 0.03 |  | 58.41 | 3.40 |
|  | Nitrate | 1.17 | 0.03 | *ab* | 0.15 | 0.00 | *a* | 12.05 | 0.32 |  | 161.80 | 13.31 | *abc* | 0.56 | 0.02 |  | 100.16 | 9.88 |  | 0.42 | 0.10 |  | 59.24 | 2.74 |
|  | Nitrate + A. brasilense | 1.10 | 0.02 | *b* | 0.15 | 0.00 | *a* | 12.73 | 0.24 |  | 162.61 | 5.83 | *abc* | 0.53 | 0.01 |  | 112.96 | 3.82 |  | 0.36 | 0.03 |  | 65.79 | 2.16 |
|  | Sulfate | 1.13 | 0.01 | *b* | 0.13 | 0.01 | *b* | 12.49 | 0.26 |  | 177.19 | 9.44 | *ab* | 0.52 | 0.00 |  | 94.25 | 1.75 |  | 0.22 | 0.01 |  | 59.10 | 1.97 |
|  | Sulfate + A. brasilense | 1.26 | 0.02 | *a* | 0.16 | 0.00 | *a* | 13.78 | 0.03 |  | 189.97 | 13.44 | *a* | 0.56 | 0.01 |  | 115.39 | 3.46 |  | 0.37 | 0.00 |  | 63.92 | 2.52 |
|  |  |  |  |  |  |  |  |  |  |  |  |  |  |  |  |  |  |  |  |  |  |  |  |  |
| **Red Rubin** | Control | 1.16 | 0.06 | *a* | 0.16 | 0.00 |  | 9.82 | 0.09 | *bc* | 109.85 | 2.24 |  | 0.54 | 0.03 | *a* | 79.16 | 0.57 | *ab* | 0.38 | 0.02 | *ab* | 58.59 | 0.79 |
|  | Control + A. brasilense | 1.01 | 0.02 | *bc* | 0.15 | 0.00 |  | 10.21 | 0.20 | *ab* | 100.05 | 8.71 |  | 0.50 | 0.01 | *ab* | 81.12 | 2.56 | *a* | 0.41 | 0.04 | *a* | 55.86 | 2.73 |
|  | Nitrate | 1.11 | 0.03 | *ab* | 0.15 | 0.00 |  | 9.03 | 0.01 | *c* | 134.40 | 25.07 |  | 0.48 | 0.02 | *ab* | 64.21 | 1.42 | *c* | 0.38 | 0.01 | *a* | 57.96 | 0.08 |
|  | Nitrate + A. brasilense | 0.97 | 0.00 | *c* | 0.14 | 0.00 |  | 9.03 | 0.30 | *c* | 102.24 | 1.22 |  | 0.46 | 0.01 | *b* | 64.54 | 0.82 | *c* | 0.26 | 0.03 | *bc* | 53.89 | 0.78 |
|  | Sulfate | 1.07 | 0.00 | *abc* | 0.14 | 0.01 |  | 10.17 | 0.24 | *b* | 126.37 | 4.53 |  | 0.49 | 0.02 | *ab* | 72.93 | 1.84 | *b* | 0.43 | 0.05 | *a* | 63.63 | 3.00 |
|  | Sulfate + A. brasilense | 1.05 | 0.01 | *abc* | 0.14 | 0.01 |  | 11.10 | 0.16 | *a* | 159.22 | 31.02 |  | 0.46 | 0.00 | *b* | 71.73 | 1.60 | *bc* | 0.19 | 0.01 | *c* | 60.77 | 3.74 |
